# Recent Advances in the Incorporation of Polysaccharides with Antioxidant and Antibacterial Functions to Preserve the Quality and Shelf Life of Meat Products

**DOI:** 10.3390/foods12081647

**Published:** 2023-04-14

**Authors:** Boutheina Ben Akacha, Monika Michalak, Basma Najar, Francesca Venturi, Isabella Taglieri, Miroslava Kačániová, Rania Ben Saad, Wissem Mnif, Stefania Garzoli, Anis Ben Hsouna

**Affiliations:** 1 Laboratory of Biotechnology and Plant Improvement, Centre of Biotechnology of Sfax, B.P “1177”, Sfax 3018, Tunisia; akachabouthaina@gmail.com (B.B.A.);; 2 Collegium Medicum, Jan Kochanowski University, IX WiekówKielc 19, 35-317 Kielce, Poland; 3 Pharmacognosy, Bioanalysis and Drug Discovery Unit and Analytical Platform, Faculty of Pharmacy, Free University of Brussels, Bld Triomphe, Campus Plaine, 205/5, B-1050 Brussels, Belgium; 4 Department of Agriculture, Food and Environment, University of Pisa, Via del Borghetto 80, 56124 Pisa, Italy; francesca.venturi@unipi.it (F.V.);; 5 Institute of Horticulture, Faculty of Horticulture, Slovak University of Agriculture, Tr. A. Hlinku 2, 949 76 Nitra, Slovakia; 6 Department of Bioenergy, Food Technology and Microbiology, Institute of Food Technology and Nutrition, University of Rzeszow, 4 Zelwerowicza St, 35-601 Rzeszow, Poland; 7 Department of Chemistry, College of Sciences at Bisha, University of Bisha, P.O. Box 199, Bisha 61922, Saudi Arabia; 8 Department of Chemistry and Technologies of Drug, Sapienza University, P. le Aldo Moro, 5, 00185 Rome, Italy; 9 Department of Environmental Sciences and Nutrition, Higher Institute of Applied Sciences and Technology of Mahdia, University of Monastir, Monastir 5000, Tunisia

**Keywords:** natural antioxidant, color preservation, antibacterial activity, bioactive compound, meat preservation, nutritional value

## Abstract

Meat and meat products are susceptible to various types of natural processes such as oxidative degradation due to their high content of protein and essential amino acids. However, finding solutions to maintain the nutritional and sensory quality of meat and meat products is unavoidable. Hence, there is a pressing need to investigate alternatives to synthetic preservatives, focusing on active biomolecules of natural provenance. Polysaccharides are natural polymers of various sources that exhibit antibacterial and antioxidant properties via a variety of mechanisms, owing to their diversity and structural variation. For this reason, these biomolecules are widely studied in order to improve texture, inhibit the growth of pathogens, and improve the oxidative stability and sensory characteristics of meat products. However, the literature has not addressed their biological activity in meat and meat products. This review summarizes the various sources of polysaccharides, their antioxidant and antibacterial activities (mainly against pathogenic food strains), and their use as natural preservatives to replace synthetic additives in meat and meat products. Special attention is given to the use of polysaccharides to improve the nutritional value of meat, resulting in more nutrient-rich meat products with higher polysaccharide content and less salt, nitrites/nitrates, and cholesterol.

## 1. Introduction

During storage, various types of alterations can affect the quality of meat and meat products, compromising their safety and consumer acceptability by reducing nutritional quality and altering sensory characteristics such as color, smell, and flavor [1,2,3]. Biochemical reactions and microbial growth may occur, leading to deterioration of the final product, affecting its biological and sensory characteristics as well as its chemical composition (saturated and unsaturated fatty acids, proteins, carbohydrates, vitamins, and pigments) [2,4]. For this reason, synthetic antioxidants are used to maintain the quality of food products by inhibiting oxidation reactions. These include sodium erythorbate, sodium ascorbate, propyl gallate, butylated hydroxyanisole (BHA), tert-butylhydroquinone (TBHQ), butylated hydroxytoluene (BHT), and curing salts such as nitrites and nitrates [1,5]. Current research, however, shows that the excessive addition and misuse of synthetic antioxidants could be associated with DNA damage, apoptosis, and carcinogenicity. “Clean label” [6] is increasingly a target in the food industry. Biopreservation is an alternative technique to extend the shelf life of meat and meat products as well as to improve their safety and microbiological quality [1,2]. Natural antimicrobial agents can enhance the taste of food, reduce the antimicrobial spectrum (enterobacteria, psychrophilic flora, etc.) [7,8,9], and minimize antimicrobial resistance [10]. Among the bioactive compounds used in the food industry, polysaccharides are receiving increasing attention. These biological macromolecules have important structural and energy functions in living organisms. In humans, polysaccharides have been demonstrated to possess significant bioactivity, such as intestinal flora regulation. Through dietary intervention, polysaccharides are a promising way to enhance immunity via regulation of intestinal microbiota. These polysaccharides, mainly of plant origin, can also exert immunomodulatory and prebiotic effects. Specifically, polysaccharides and products derived from microbiota can influence the balance between strengthening and suppressing the immune system modulating the release of pro-inflammatory cytokines [11]. In addition, many other biological activities have been demonstrated such as coagulation and antiviral, hematopoietic, anti-inflammatory and immunological effects [9,12]. Polysaccharides have also been shown to exhibit antitumor activity; for example, *Astragalus Membranaceusa* polysaccharides successfully inhibited the growth of solid tumors of H22 hepatocarcinoma transplanted in BALB/c mice, decreased serum IL-10 levels, and promoted TNF-a, IL-2, and IL-12 secretion [13].

Polysaccharides are characterized by the presence of different functional groups, which makes them suitable for the preparation of various bio-nanostructures. In particular, the application of nanotechnology in the food sector has led to the development of polysaccharide-based nanostructures, obtaining excellent results both in terms of increased food quality and shelf-life extension as well as better protection of food from environmental influences such as heat, light, oxygen, enzymes, dust and gas emissions. In fact, thanks to the excellent characteristics of polysaccharides, including their high biodegradability and low toxicity, different types of nanostructures such as nanoparticles including nanospheres and nanocapsules, nanocomposites subdivided into graphene/carbon-nanotubes, metal oxide-based hybrid materials, dendrimeric nanostructures and metal-polysaccharide hybrids have also been developed to make food packaging mainly in the form of edible coatings and films. Furthermore, the latest applications for the creation of eco-friendly packaging systems also include the use of hemicelluloses, marine polysaccharides, and bacterial exopolysaccharides. In conclusion, the main advantage of using these modern applications consists of the partial or total elimination of conventional packaging materials, thus reducing the use of plastic material [14,15].

The purpose of this study is to provide comprehensive knowledge regarding the application of polysaccharides in the preservation of meat and meat products as natural preservatives during refrigerated storage. It also aims to gather findings from studies on the antioxidant and antimicrobial activities of polysaccharides from various natural sources.

## 2. The Structure of Polysaccharides

**Figure 1:**
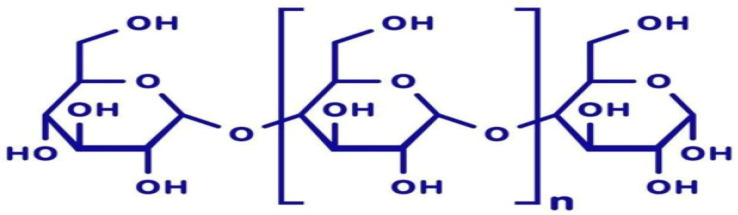
Basic structure of polysaccharides [16].

Polysaccharides, also called glycans, are polymeric carbohydrate molecules made up of long chains of monosaccharide units linked together with *O*-glycosidic bonds. The most important compounds of this class, cellulose, starch and glycogen, are all polymers of glucose (Figure 1). 

It is estimated that more than 90% of the carbohydrate mass in nature is in the form of polysaccharides. Naturally occurring polysaccharides have specific structural characteristics due to different intrinsic properties such as molecular weight, the type of monosaccharides that constitute them, the configuration (α or β), or the degree of branching. All of these structural properties are responsible for the functional properties of polysaccharides, including their solubility. Homopolysaccharides, also known as homoglycans, are made up of the same monosaccharides, while heteropolysaccharides (heteroglycans) are made up of different monosaccharides [17]. The most frequent constituent of polysaccharides is d-glucose; however, d-fructose, d-galactose, l-galactose, d-mannose, l-arabinose, and d-xylose are also common. Some chemical modifications to polysaccharides, such as solvation and phosphorylation, can efficiently modify their biological properties [18]. Given that the activity of polysaccharides takes place mainly in aqueous solutions, it is essential to understand the mechanisms that regulate solubility [19]. In this context, molecular weight clearly plays a fundamental role as larger molecules with high molecular weights possess lower solubility [20]. Charged polysaccharides possess both negatively and positively charged groups. The presence of charged groups improves the solubility of polysaccharides. There is a significant difference in terms of solubility between linear and branched polysaccharides; the former are mostly insoluble in water, while the latter are more soluble. Similar to branching effects, the presence of some hydrophobic groups, such as O-Ac and O-Me, could affect the solubility of polysaccharides [21].

## 3. Sources and Characteristics of Polysaccharides

**Figure 2:**
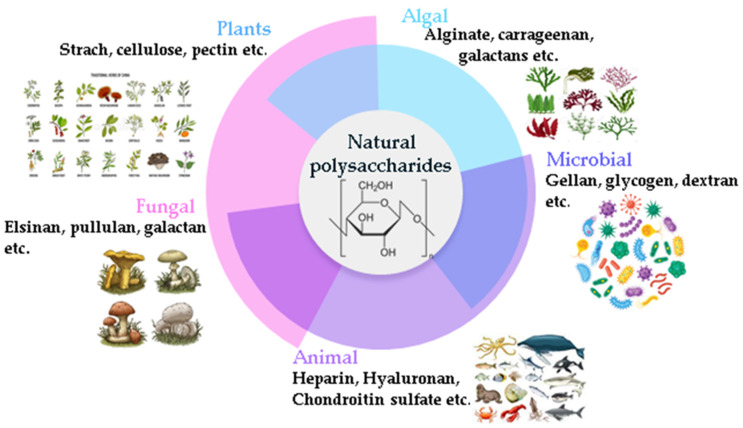
Classification of natural polysaccharides based on their source.

The properties of polysaccharides depend mainly on the type of monosaccharides, their links, and their molecular weights. Due to the diverse uses of polysaccharides in various industries, as well as their high efficiency, convenience, low cost, and environmental impact, interesting methods have been developed to extract and purify polysaccharides from renewable sources such as plants, algae, microorganisms, and animals [9,16,22,23] (Figure 2).

Based on their functionality, storage, and structure, these polysaccharides are classified into two categories: (1) polymers that are a component of plants’ energy stores and (2) polymers that are a constituent of cell walls, giving the plant rigidity and flexibility. The Food and Agriculture Organization of the United Nations (FAO) defines a variety of plant polysaccharides as dietary fibers, mainly cellulose, pectins, gums, and oligosaccharides. The most important of these are cellulose and pectins [13,24]. In addition to functional polysaccharides from plants, animal-derived polysaccharides also play an essential role in the composition of tissues. Furthermore, these biological macromolecules play a structural and storage role in animals as part of tissues and cell matrices [25]. They are considered natural biopolymers due to their biodegradability, biocompatibility, non-toxicity (for example, heteropolysaccharides of *Lobularia maritima* with LD_50_ > 250 mg/kg [9]), and non-antigenicity (the antigenicity of collagen is assumed to be non-existent with 3% anti-implant antibodies after injection) [25]. Owing to these properties, they possess biomedical, pharmaceutical, and food applications [26]. As structural compounds, energy storage, and in the form of mucopolysaccharides, polysaccharides are the most abundant macromolecules in the structure of algae [27]. Polysaccharide content varies depending on the species, ranging from 4% to 76% of the dry weight of the algae. For example, green algae contain lignin, cellulose, and hemicellulose; brown algae contain only cellulose; and red algae are composed of dietary fibers [28]. 

As with polysaccharides from other organisms, different classifications of polysaccharides from macroalgae are encountered in the literature: structural and matrix polysaccharides, anionic and neutral polysaccharides, and sulfated and non-sulfated polysaccharides. Microorganisms and macromycetes are also important sources of natural polysaccharides [27,28].

Bacterial polysaccharides are natural biopolymers consisting of monosaccharide chains. They can be produced in two ways: extracellularly and intracellularly. Depending on their cellular localization, some play a reserve role and are localized in the cytoplasm, while others are macromolecules constituting walls [29]. Some species of bacteria excrete polysaccharides in their extracellular environments, which may or may not be associated with plasma membranes. Depending on the type of monosaccharide chain, polysaccharides have rheological, biological, and physicochemical properties; therefore, these molecules are valued for their thickening, stabilizing, and gelling properties. In addition, they exhibit antiviral, antitumor, anti-inflammatory, and antimicrobial activities [30]. In terms of industrial applications, the greatest benefit of these polysaccharides is that they can be produced in a bioreactor free from climatic, ecological, and political constraints [31]. Indeed, researchers have discovered five distinct natural sources of polysaccharides, each possessing unique structural and functional characteristics, making them the most ubiquitous natural polymer on Earth [31]. Due to their diverse biological properties, such as antioxidant and antimicrobial actions, polysaccharides have a wide range of useful applications.

Regarding the extraction method for bacterial polysaccharides, two different main approaches can be followed: (i) enzymatic syntheses in which several isolated enzymes can be utilized in combination to produce the target oligo/polysaccharide via cascade reactions and (ii) a cell factory strategy in which an engineered microbial host is enabled to produce oligo/polysaccharides via a heterogeneous biosynthesis pathway for which neither purification of related enzymes nor construction of cofactor regeneration systems is required [32].

As recently reviewed by Lin et al. [28], polysaccharide properties and related polysaccharide-derived hydrogels are deeply affected by purification processes as well as by extraction conditions.

## 4. Antioxidant and Antimicrobial Activities of Polysaccharides

Modern lifestyles contribute to deficiencies in various bioactive compounds, including components that can protect against the adverse effects of free radicals and oxidative stress [33,34,35,36,37], thus increasing the risk of various diseases. 

Hence, natural sources of antioxidants are sought to compensate for deficits in diets. In addition to their nutritional role, antioxidants play an important role in the preservation of food by inhibiting oxidation [2,5,10,38]. Recent research results indicate that polysaccharides from natural products have many beneficial therapeutic effects and health properties [39,40,41]. The antioxidant activity of polysaccharides has been evaluated through various methods, e.g., 2,2-diphenyl-1-picrylhydrazyl (DPPH), ferric reducing antioxidant power (FRAP), metal chelating activity, 2,2′-azino-bis(3-ethylbenzothiazoline-6-sulfonic acid (ABTS), hydroxyl radical scavenging activity, and the β-carotene-linoleate bleaching assay [9,42,43,44]. In addition to their antioxidant properties, polysaccharides present interesting antibacterial activity, which has been evaluated through an agar diffusion test and the determination of the minimum inhibitory concentration (MIC) [45]. Table 1 summary the results of antioxidant and antimicrobial activities of polysaccharides previously reported in the literature. 

### 4.1. Plant Polysaccharides

Recent studies have shown that plant polysaccharides offer a variety of biological benefits, including antioxidant and antibiotic activities. Polysaccharides derived from edible resources are safer and more effective, with fewer side effects than other sources. They are also more readily available and less expensive. Thus, most bioactive polysaccharides from various plants are important materials for food and therapeutic applications [13]. For example, polysaccharides isolated from olive leaves have shown strong DPPH scavenging activity (IC_50_ = 34.80 µg/mL) as well as significant reducing power and β-carotene bleaching inhibition activity [46]. This polysaccharide exhibits important antibacterial activity against several pathogenic strains considered resistant to standard antibiotics such as *S. enterica* and *E. coli* [44].

Han et al. [22] evaluated the antioxidant and antibacterial potentials of polysaccharides extracted from *Broussonetia papyrifera* fruits and showed important hydroxyl radical scavenging activity, ferric reducing activity power, and antibacterial activity against four pathogenic strains. Studies by Meng et al. [23] revealed that a water-soluble polysaccharide fraction from *Diaphragma juglandis* fruit exhibited significant antioxidant and antibacterial activities. Other studies have shown that two fractions of polysaccharides extracted from *Malva aegyptiaca* presented a wide spectrum of antibacterial activity (especially against gram-positive bacteria) and also displayed important antioxidant activity [47]. The studies cited show that polysaccharides may potentially be used as natural antioxidants and bacteriostatic agents in the food or medical industries. Plants have been utilized to treat a variety of disorders in the traditional medicines of many nations, including traditional Chinese medicine and the phytomedicines of Western nations [13]. Modern experiments have found that in addition to such plant metabolites as flavonoids, saponins and alkaloids, polysaccharides play an important role, owing to their various pharmacological effects.

### 4.2. Fungal Polysaccharides

Fungal polysaccharides are found in cell walls or formed by energy processes in edible fungi and yeasts [56,57]. These are polymeric molecules with linear and branched structures composed of homopolysaccharides and heteropolysaccharides that exhibit various biological properties [57,58]. Many scientific studies, reports, and patents refer to the possibility of their use in various fields. For example, Liu et al. [48] reported that mycelial polysaccharides from *Catathelasma ventricosum* modified with carboxymethylation exhibited an excellent inhibitory effect on *Escherichia coli*, *Salmonella typhimurium*, *Staphylococcus aureus*, and *Bacillus subtilis*. The findings of earlier studies indicate that carboxymethylated polysaccharides from *C. ventricosum* can be used as potential alternatives to antibiotics as antibacterial agents. Moreover, carboxymethylated polysaccharides showed strong antioxidant activity determined by their DPPH radical scavenging activity, reducing power, and metal chelating activity [48]. In the same context, the antioxidant and antibacterial activities of intracellular zinc polysaccharides from *Grifola frondosa* SH-05 have been evaluated [49]. The results indicate that IZPPS and IPS exhibit important antioxidant properties by scavenging hydroxyl and DPPH radicals, as well as show Fe^2+^ chelating activity. In addition, IZPS showed potential antibacterial activity against foodborne pathogens. The exact antibacterial mechanism of polysaccharides is not yet known. It is suggested that polysaccharides can disrupt bacterial cell walls and cytoplasmic membranes, causing degradation and leakage of essential molecules [59].

In addition to antioxidant and antimicrobial properties, medicinal mushrooms are reported to exhibit anticancer, antioxidant, antimicrobial, hepatoprotective, antineurodegenerative, antidiabetic, antiangiogenic, and hypoglycemic activity [48,49,59,60,61,62]. According to the authors, polysaccharides of fungal origin have distinctive structures (the spatial conformation of the molecule, degree of branching, and molecular mass) that affect their biological activities. These polysaccharides have demonstrated in vitro effectiveness against pathogenic microbes resistant to conventional antibiotics. In addition to their potent antibacterial activity, they are also natural antioxidants with a variety of applications.

### 4.3. Algal Polysaccharides

Polysaccharides are the most abundant macromolecules in algal structures and exist as structural and energy storage mucopolysaccharides. Polysaccharides account for 4–76% of the dry weight of algae [27,63]. Algal polysaccharides are of increasing interest due to their excellent physical properties (gelation, thickening, and stabilization) as well as their beneficial biological activities, including anticoagulant, antimicrobial, antithrombotic, antioxidant, antiviral, and anti-inflammatory effects [42,64,65,66]. For example, fucoidan polysaccharides (mainly consisting of fucose) isolated from brown algae *Spatoglossum asperum* exhibited high antioxidant and DPPH scavenging activities with a significant IC_50_ of 76.80 µg/mL [37]. Thus, fucoidan exhibited the maximum reducing power at 50 mg/mL (42.63%). These results are in line with previous reports stating that the total antioxidant activity of isolated fucoidan is somewhat similar to that of fucoidans isolated from *Padina tetrastomatica* and the sulfated polysaccharide isolated from *Pterocladia capillacea* [51,67].

The antibacterial activity of fucoidan isolated from *Spatoglossum asperum* has been evaluated against *A. hydrophila* using a confocal laser scanning microscope (CLSM 710). Interestingly, the number of stained cells gradually decreased when the bacteria were treated with different concentrations of fucoidan, showing the effective antibacterial efficacy of this polysaccharide. Sellimi et al. [52] investigated new polysaccharides conjugated to proteins and polyphenols (CBG) isolated from the Tunisian alga *Cystoseira barbata*. Their antimicrobial activity was evaluated against five fungal and eight bacterial strains involved in food poisoning. Among the gram-positive bacteria tested, *S. aureus* proved to be the most sensitive to CBG (IZD = 19 mm, MIC = 10 mg/mL). Research results revealed that CBGs can be used as antibacterial agents against *S. aureus*, which causes vomiting, diarrhea, and abdominal cramps and also spoils raw meat, poultry, ham, dairy products, salads, and shrimp [68]. 

Moreover, research results revealed that polysaccharides conjugated with protein and polyphenols displayed perceptible antioxidant activities. Owing to this structural specificity, polysaccharides exhibit high free radical scavenging capacities. The above findings indicate that *Cystoseira barbata* glycoconjugates can provide a new safe and environmentally friendly means of food biopreservation [52]. 

### 4.4. Animal Polysaccharides

Some research has examined the antioxidant and antimicrobial properties of polysaccharides derived from animals [69,70,71,72,73]. Jridi et al. [53] discovered that polysaccharides extracted from cuttlefish skin and muscles contain high amounts of sulfate and uronic acid, indicating that they have possess antioxidant and antibacterial potentials. These active molecules display antioxidant properties by inhibiting the propagation of the radical chain reaction, donating hydrogen or electrons to free radicals, or chelating iron. The antimicrobial activity of these polysaccharides has been evaluated using the agar diffusion method. The inhibition zones showed diameters greater than 22.7–15.7 mm against *Enterobacter* sp. However, *B. cereus* was the most resistant strain with the weakest inhibition zone diameter. The MIC results also indicate that cuttlefish polysaccharides exhibit more potent antibacterial activity against gram-negative bacteria [53]. Therefore, these polysaccharides can be considered natural preservatives against food-borne pathogens and are likely useful in food production industries and for the protection of human health [74,75]. Similarly, sulfated polysaccharides of the common smooth hound studied by Abdelhedi et al. [54] showed significant antibacterial activity with inhibition zones of about 3 cm in diameter for *M. luteus* and *K. pneumoniae*. This antibacterial activity was slightly more potent against gram-negative bacteria. 

The ability of polysaccharides to disrupt cell membranes is enhanced by the presence of sulfate groups within the polysaccharides’ structures, resulting in potent antibacterial activity. Antioxidant activity results were also significant, indicating that these polysaccharides are natural antioxidants. The above results indicate that they can therefore be considered natural preservatives against food-borne pathogens [54].

### 4.5. Microbial Polysaccharides

Microbial polysaccharides are water-soluble carbohydrate polymers with high molecular weights produced by various bacteria [76,77,78]. Based on their rheological characteristics [79,80], microbial polysaccharides are used as binders, coagulants, emulsifiers, film formers, gelling agents, lubricants, stabilizers, thickeners, and suspending agents [29,78,81]. Recent improvements have focused on the potential applications (in the cosmetic, medical, food, pharmaceutical, and other industries) of these polymers for human use [79,81,82,83]. The antioxidant and antibacterial activities of *Pleurotus eryngii* polysaccharides (PEPS) and *Streptococcus thermophilus* ASCC 1275 exopolysaccharides (ST1275 EPS) were studied by Li and Shah [28]. The antioxidant activities of this polysaccharide were determined through DPPH, superoxide, and hydroxyl radical scavenging tests and through the FRAP assay. The results indicate that sulfated modification improved the activity of PEPS and ST1275 EPS significantly in all four antioxidant activity tests. 

Furthermore, the researchers showed that these polysaccharides are effective against three pathogenic species—*E. coli*, *S. aureus*, and *L. monocytogenes.* These results indicate that microbial polysaccharides can be used in various fields, mainly in the food industry as natural preservatives. It is interesting to look at polysaccharides as biocontrol agents to limit the formation of biofilms caused by pathogens. Mahdhi et al. [55] investigated the physicochemical properties of an exopolysaccharide (EPS) isolated from *Lactobacillus plantarum*, as well as its effect on biofilm formation. The results revealed that this polysaccharide exerts an antibiofilm effect. 

Moreover, this polysaccharide exhibited noticeable antibacterial activity with no cytotoxic effect as well as significant potential to scavenge DPPH radicals and inhibit linoleic acid peroxidation [55]. Taking into account all these biological properties, EPS can be considered a potential prebiotic agent that may be used in the creation of novel food additives as well as in new therapeutic approaches for the treatment of bacterial infections linked to biofilms and the reduction of biofilm formations on indwelling medical devices [84,85,86,87].

This leads to the conclusion that animal polysaccharides are natural biopolymers with a variety of bioactive properties that can be exploited by the food industry as alternatives to petroleum-based polymers and synthetic preservatives.

## 5. Application of Polysaccharides as Natural Preservatives in Meat and Meat Products

**Figure 3:**
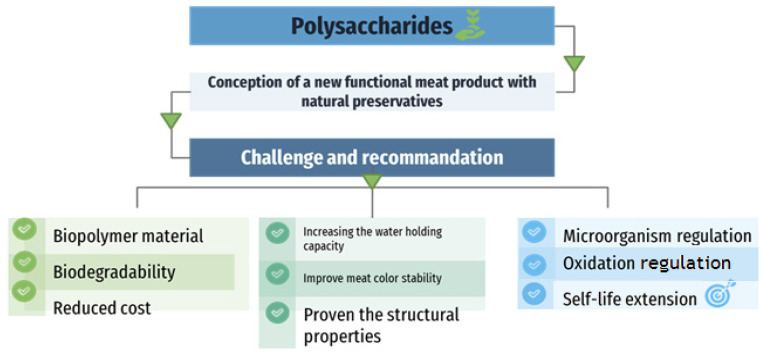
Challenges and recommendations for the meat and meat-products industry’s implementation of polysaccharides as preservatives.

Research in the meat and meat-products industry is focused on finding effective innovative techniques to prevent the negative effects associated with the mass use of artificial preservatives. Given their potential activities [88,89,90,91], polysaccharides have become the subject of several types of research due to their potential as natural food additives and active ingredients with anti-microbial and antioxidant properties (Figure 3) [14,30,92]. 

In addition, they could represent a successful breakthrough by replacing synthetic antioxidants, which are still questionable for their toxicological effects [93,94]. Table 2 presents recent results in this area.

### 5.1. Beef

**Figure 4:**
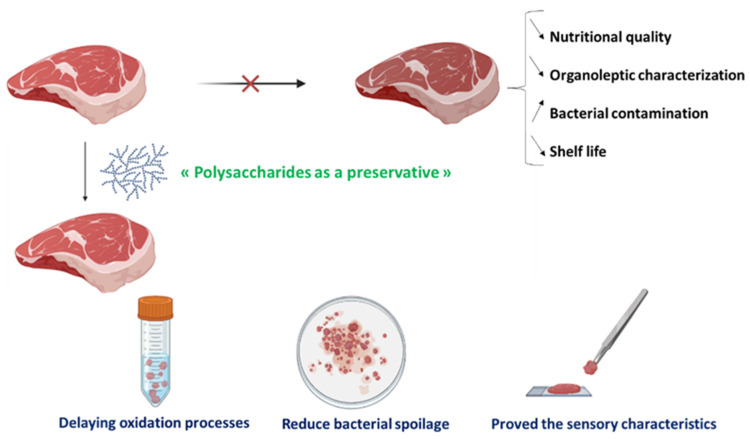
Incorporation of polysaccharides in beef for oxidative stability, reduction of bacterial contamination, and improved organoleptic quality through time.

Hamed et al. [48] reported that incorporating crude polysaccharides (Figure 4) from the outer shell of pistachios into beef has several advantages in the storage of ground beef. From the sixth day of storage, samples supplemented with polysaccharides (1% and 2%) showed significantly lower levels of thiobarbituric acid reactive substances (TBARS) than control samples. At the end of storage, samples prepared with BHT (0.5%) and with crude polysaccharides (1% and 2%) had TBARS values of 0.440, 0.321, and 0.228 mg MDA/kg, respectively. These results suggest that pistachio polysaccharides may act as antioxidant compounds to retard lipid oxidation during storage. The promising ability to protect beef from lipid oxidation and prolong product shelf life may be due to the antioxidant activity of polysaccharides from raw pistachio shells. The red color of the meat is due to oxymyoglobin and indicates its freshness. Therefore, the retail shelf life of chilled beef is limited by the formation of brown metmyoglobin, which is the oxidized form of oxymyoglobin [98]. The color attributes of the samples were expressed as lightness (L*) and redness (a*). Instrumental color measurements revealed that the addition of crude polysaccharides affected the colors of the samples. A decrease in lightness was also noted for all samples during the storage period. In addition, at the end of storage, the sample treated with 2% crude polysaccharides had a higher L* value than the other samples. These results indicate that crude polysaccharides should be developed as functional and bioactive components for the food and nutraceutical industries.

Using the same strategy of incorporating polysaccharides into meat (Figure 4), given their interesting antioxidant and antimicrobial activities, Kallel et al. [96] used them as a natural preservative in beef patties during refrigerated storage. The authors prepared meat samples using 2% and 4% formulations in order to extend the shelf life of the meat while preserving its best characteristics. The lipid oxidation measurement, microbial measurement, instrumental color measurement, and sensory properties were evaluated. The results revealed that the TBARS values of all beef cuts treated with 2% and 4% GSP (grape skin and seed pomace) were lower than those of the two control cuts. This protective effect of GSP against lipid peroxidation found by Kallel et al. [96] can be explained by the presence of antioxidant compounds in the by-product. The data showed that the shelf life of the samples would be 3 days, while, for the samples in the GSP and BHA groups, it could be extended from 3 to 6 days of storage, respectively. The results obtained for color deterioration during refrigerated storage of beef patties showed that the GSP group possessed better color than the negative control. This could be explained by the antioxidant effect of polysaccharides, which retarded the formation of metmyoglobin when included at 2% (*w*/*w*). All of these results were finally confirmed through sensory analysis, which showed that the incorporation of GSP in ground-beef patties could improve sensory attributes and extend shelf life during refrigerated storage.

A study by Ben Hlima et al. [97] found that the addition of various concentrations of sulfated exopolysaccharides from *Porphyridium cruentum* (EPS) to ground beef significantly reduced primary and secondary lipid oxidation, lowering metmyoglobin levels compared to control samples. In addition, the accumulation of carbonyl groups was reduced. Microbiological analysis showed that the addition of EPS significantly improved the quality of raw ground beef during refrigerated storage. Thus, EPS could improve the microbiological quality and oxidative stability of ground beef for 14 days at 4 °C. Therefore, EPS can be successfully used to extend the shelf life and improve the health benefits of refrigerated meat products.

### 5.2. Beef and Turkey Sausages

Many studies have evaluated the potential action of various polysaccharides to inhibit oxidative reactions and microbial growth in meat products and meat. In this context, Trabelsi et al. [99] investigated the technological applications of natural polysaccharides in food-product formulations. In particular, the substitution of vitamin C with EPS-Ca6 for cooked beef sausage was evaluated. After 4 days, sausages containing 0.0625% and 0.125% EPS-Ca6 had significantly lower TBARS levels (0.29 and 0.26 mg MDA eq/kg, respectively) compared to control samples (0.35 mg MDA eq/kg). These results revealed that the addition of EPS-Ca6 could delay lipid peroxidation during refrigerated storage. 

The color of meat products depends on the amount of myoglobin and its chemical forms (oxymyoglobin (OxyMb), metmyoglobin (MetMb), etc.). Discoloration of meat affects its appearance, which can be explained by the conversion of OxyMb to MetMb. At the end of refrigerated storage, EPS-Ca6 at 0.0625% and EPS-Ca6 at 0.125% exhibited the highest oxidation of OxyMb, at 24.12% and 25.98%, respectively. According to this study, EPS-Ca6 could act as a good cross-linker by providing the second electron necessary for the reduction of oxygen in the oxidation of OxyMb to MetMb and oxygen radicals (hydrogen peroxide or superoxide). It can be concluded that EPS-Ca6 is a promising candidate for use as a natural and safe antioxidant as well as a functional ingredient in several food products. Hamzaoui et al. [100] formulated a new beef sausage with polysaccharides extracted from green algae *Chaetomorpha linum* (PS) added at different concentrations (0.05%, 0.125%, and 0.25%) and compared it with two controls (the positive control group was supplemented with 0.125% vitamin C, while the negative control group was not supplemented with vitamin C or PS).

For sausages formulated without antioxidant supplementation, the data showed a significant reduction in pH values during storage compared to the PS-treated formulations. The addition of PS at a concentration of 0.25% in the sausage formulation caused an increase in moisture content to 2.1% at the end of the storage period (which can be attributed to the hydrophilic nature of the fiber) and led to a reduction in redness values compared to the control sausages. The results indicated that PS was effective in reducing lipid oxidation during storage; it demonstrates high efficiency when compared to the standard sample with respect to MetMb, TBARS, and heme iron levels. Conjugated dienes and free fatty acids showed the same tendency. In addition, due to the uptake of PS, the microbial community was reduced, thus demonstrating its potential as a bioactive dietary additive.

In the same context, Ktari et al. [101] studied the effect of a fenugreek water-soluble polysaccharide (FWSP) on oxidative processes in beef sausages during refrigerated storage (4 °C). The findings demonstrated the effectiveness and utility of FWSP as an antioxidant that preserves the storage stability of beef sausages and can provide a substitute for vitamin C, which is currently used as an antioxidant in industrial processes. The findings demonstrated important inhibition of lipid and myoglobin oxidation. Another study showed that a new water-soluble polysaccharide isolated from *Anethum graveolens* seeds (AGP1) could be used in turkey sausages as a preservative instead of ascorbic acid [102]. The results showed that this preservation method increased bacterial stability during cold storage at 4 °C for 12 days, decreased lipid peroxidation, and maintained pH and color. 

In another study, Luo et al. [103] examined the effect of *Spirulina platensis* polysaccharides (SPP) at concentrations of 0.1%, 0.25%, and 0.5% in Chinese-style sausages on lipid peroxidation and microbiological and sensory properties during 24 days of storage at 4 °C. The results showed that the addition of SPP caused a dose-dependent decrease in pH and prevented the color change (a*), which was probably linked to the low acidity of fucoidan in this polysaccharide [49,104]. Moreover, SPP could exert an antioxidant effect to protect against iron oxidation during storage at 4 °C. As a result, it was shown to improve the sensory characteristics (aroma, flavor, and overall acceptability) of the product, so it can be added as a natural antioxidant and sensory enhancer to Chinese-style sausages.

The studies present in the literature on functionalized polysaccharides in meat and meat-based products, are relatively recent. As reported by the authors, polysaccharides could influence the sensory attributes of the final product by improving its texture, smell, and by preserving its color, an effect closely related to the antioxidant properties of polysaccharides. Furthermore, in most of the cited studies, it is also reported that polysaccharides extend the shelf life of products by preventing contamination by pathogenic microorganisms thanks to their antibacterial action. Lastly, further studies followed by practical applications should be conducted to implement the industrial use of polysaccharides in the food sector in order to commercialize meat products with added polysaccharides as natural preservatives.

### 5.3. Poultry Sausage, Chicken Breast, and Pork Patties

For the purpose of enhancing the properties of chicken sausage, Andrès et al. [105] used whey protein concentrates and hydrocolloids (xanthan gum/guar in a 3:7 ratio). The results showed that increasing the concentration of the formulation improved the textural properties of the sausage (by decreasing hardness), its color (by modifying the lightness and redness), and its microstructure (by increasing cohesion and decreasing the granular matrix). In conclusion, the low-fat sausages were sensorial acceptable, and the added ingredients enhanced their functional properties.

Another study found that a blackberry polysaccharide can significantly improve the elasticity, flavor, and color of chicken-breast meat [106]. Breast meat was marinated for 24 h in different concentrations of isolated blackberry polysaccharide (1 g/kg and 3 g/kg) at a material/liquid ratio of 1:3. The results showed that the addition of the blackberry polysaccharide could significantly improve the hardness of chicken breast and the conversion of free water to bound water after one hour of cooking. This effect might be linked to the composition of polysaccharides (95.44% glucose, 2.01% arabinose, 1.81% galactose, and 0.74% glucuronic acid), which makes them valuable as natural preservatives.

Latou et al. [107] investigated the combined effect of chitosan and modified-atmosphere packaging on the shelf life of chicken breast fillets. The results showed that shelf life was extended by 9 days, with preservation of microbiological parameters (total viable counts, *Pseudomonas* spp., lactic acid bacteria, and enterobacteria) and protection against exponential variations of physicochemical parameters (headspace gas composition, pH, color, and thiobarbituric acid test). Finally, improvement of sensory parameters (odor and taste) allowed for the monitoring of the tested samples for up to 14 days, owing to the antioxidant and antimicrobial activities of chitosan.

The use of polysaccharides as food additives is gaining popularity due to their wide range of functional properties, including the preservation and improvement of pork patties [107]. For example, brown seaweed extract (*Laminaria digitata*) containing a significant amount of laminarin and fucoidan was used to improve the quality and shelf life of fresh and cooked ground pork patties. The authors demonstrated that this treatment reduced the surface redness (a* values) of fresh patties in a concentration-dependent way with high pro-oxidative activity of lipids in fresh patties and substantially reduced lipid oxidation in cooked patties. The sensory panelists favored pork patties with 0.01% brown seaweed (*Laminaria digitata*) extract. The presence of laminarin and fucoidan makes refined and purified seaweed extracts suitable for use in functional meat products.

**Figure 5:**
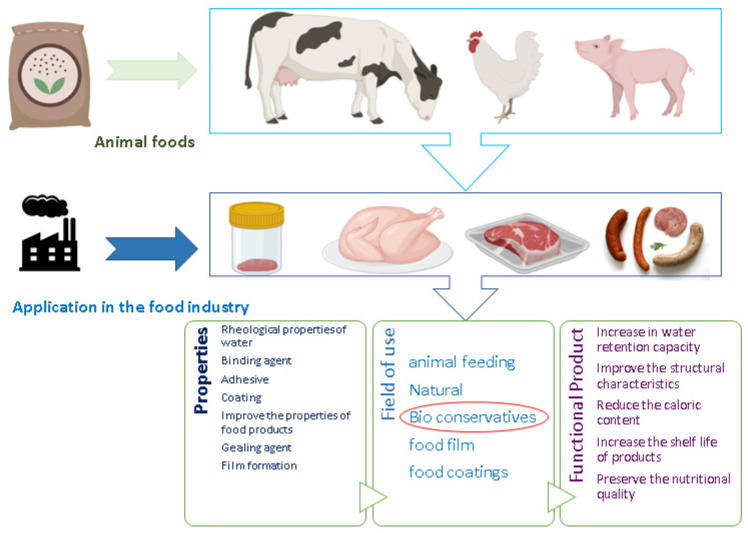
Functional meat products consisting of polysaccharides.

Overall, studies have shown that polysaccharides used as active ingredients improve the properties of meat even of different origins (pork, beef, or chicken) and that they can be used as preservatives in meat and meat products (Figure 5). However, the structural and functional characteristics of polysaccharides provide a wide range of application methodologies for these polymers, both by direct incorporation into the product, for packaging, and by synthesis of a food film. These different techniques have further demonstrated the efficacy of polysaccharides for the improvement of the functional quality of foods, i.e., as stabilizing agents, thickeners, emulsifiers, and humectants. The latter proved to be invaluable and can be used in food industries as additives.

## 6. Final Considerations

Based on their functionality and biological activity, polysaccharides of various origins are used for the preservation of meat and meat products. The literature data underline their capacity to improve the properties of food (acceptability, smell, appearance, and texture), to inhibit the growth of pathogenic bacteria (enterobacteria, psychrophilic flora, and total mesophilic flora), and to exert a pro-oxidant effect against the oxidation of lipids and proteins, and therefore against the discoloration of these products, resulting in longer preservation and freshness of meat and meat products. The research results cited in this review indicate that polysaccharides possess antibacterial and antioxidant properties depending on their origins, structures, and compositions. Innovations in meat-product technology may influence the production of more nutrient-rich meat products containing more polysaccharides to respond to changes in the eating habits of consumers who are increasingly concerned about their health and prioritize the consumption of more natural foods with fewer preservatives. There is a need for further research on the use of polysaccharides as valuable bioactive ingredients in meat products to improve their nutritional value in connection with the beneficial effects of these active compounds on human health and the absence of secondary effects.

## Figures and Tables

**Table 1:** Antioxidant and antimicrobial activities of polysaccharides previously reported in the literature.

Polysaccharide	Main Sources	Antioxidant Activity		Antimicrobial Activity			References
		Method	Values	Target Microorganism	MIC (mg/mL)	ZI (mm)	
*Broussonetia papyrifera* polysaccharide (BPP)	*Broussonetia papyrifera* 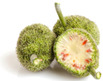	DPPH assay IC_50_ (mg/mL)	0.54–0.84	*E. coli*	0.3–1 0.25–1	9.71–11.5	[22]
		Hydroxyl radical scavenging activity IC_50_ (mg/mL)	1.28–2.09	*P. aeruginosa*	0.25–1	7.39–12.77	
		Ferric—reducing activity power (mmol/L)	0.37–0.74	*B. subtilis*	0.3–4	6.00–9.84	
		Erythrocytehemolysis (%)	60.09–79.69	*S. aureus*	0.25–1	7.06–13.40	
Olive trees polysaccharide (OLP)	*Olea europaea* 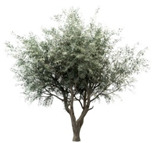	DPPH assay IC_50_ (µg/mL)	34.80	*B. cereus* *M. luteus*	-	10 21.5	[46]
		β-carotenelinoleate bleaching assay (%)	59.51–500	*S. enterica* *E. coli*	-	23.5 10.5	
		Reducing power assay (µg/mL)	106.31	*Enterobacter* sp. *K. pneumonieae*	-	9.5 -	
A water-soluble polysaccharide fraction (DJP-2)	*Diaphragma juglandis* 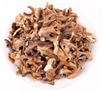	DPPH assay IC_50_ (mg/mL)	1.068	*E. coli* *P. aeruginosa*	-	8.22–14.85 8.42–15.31	[23]
		ABTS assay IC_50_ (mg/mL)	0.649	*S. aureus*	-	9.11–15.97	
		Hydroxyl radical scavenging activity	0.909	*E. faecalis*	-	8.12–14.35	
Polysaccharides extracted via precipitation with cetylpyridinium chloride (P1) or ethanol (P2)	*Malva aegyptiaca* 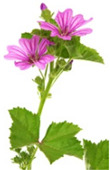	(Fe^2+^) chelating activity IC_50_ (mg/mL)	1.15–3.30	*S. aureus* *M. luteus*	-	7.5–18.5 20.0–10.0	[47]
		(Fe^3+^) reducing antioxidant power (FRAP) EC_50_ (mg/mL)	1.22–4.5	*B. cereus* *E. coli*	-	19.5–8.5 18.5–13.5	
		β-carotene bleaching inhibition capacity IC_50_ (mg/mL)	1.56–2.74	*K. pneumoniae*	-	25.0–19.5	
		DPPH assay IC_50_ (mg/mL)	1.94–3.57	*S. enterica* *S.typhi*	-	12.5–5.0 17.5–10.5	
Mycelial polysaccharides modified via carboxymethylation (cmCVP-1Ss)	*Catathelasma ventricosum* 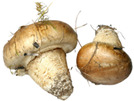	DPPH assay	3.73–18.40	*E. coli* *S. typhimurium*	2.14–10.86 2.85–4.76	3.55–17.60 4.40–8.53	[48]
		Reducing power EC_50_ (mg/mL)	1.04–14.64	*S. aureus*	1.78–6.89	4.01–12.22	
		Metal chelating activity EC_50_ (mg/mL)	2.85–8.95	*B. subtilis*	2.25–4.63	3.75–9.05	
Intracellular zinc polysaccharides (IZPS)	*Grifola frondosa SH-05* 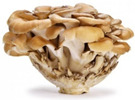	Hydroxyl radical-scavenging assay EC_50_ (mg/mL) Reducing power assay At 1000 mg/mL DPPH assay EC_50_ (mg/mL) Superoxide radical-scavenging activity EC_50_ (mg/mL) Hydrogen peroxide-scavenging activity at 1000 mg/mL Ferrous ion chelating activity at 1000 mg/mL	203.7–510 0.59–0.38 211.2 525.27 90.31–95.23 27.09–50.92	*E. coli* *S. aureus* *B. megaterium* *L. monocytogenes*	5–1.25 2.5–0.625 10.0–2.5 5–2.5	13.2–30 18.1–39.7 14.6–26.3 15.5–28.6	[49]
Fucoidan	*Spatoglossum asperum* 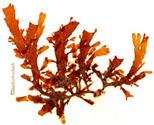	DPPH assay IC_50_ (µg/mL)	76.80	*A. hydrophila*	-	32	[50]
		Reducing power assay (%) at 500 mg/mL	42.63				
		Total antioxidant activity IC_50_ (µg/mL)	89.81				
Sulfated polysaccharides (SPs)	*Pterocladia capillacea* 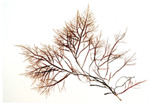	DPPH assay IC_50_ (µg/mL)	530–1104	*S. aureus*	-	7–9.2	[51]
		Hydrogen peroxide scavenging assay IC_50_ (µg/mL)	1093–8143	*E. coli*	-	8	
Polysaccharides conjugated to proteins and polyphenols (CBG)	*Cystoseira barbata* 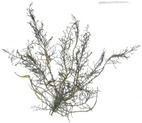	DPPH assay EC_50_ (µg/mL)	11.70	*S. aureus* *B. cereus*	10 20	19 11	[52]
		Iron (III) to iron (II)-reducing activity EC_50_ (µg/mL)	51.22	*E. faecalis* *M. luteus*	20 20	11 11	
		Ferrous ion-chelating activity EC_50_ (µg/mL)	40.31	*E. coli* *P.aeruginosa* *S. enterica*	- -	- - -	
		Hydroxyl radical-scavenging activity EC_50_ (µg/mL)	11.39	*K. pneumoniae*	40	8	
Polysaccharides extracted from cuttlefish skin (CSP) and muscles (CMP)	Cuttlefish (*Sepia officinalis*) 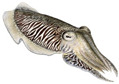	DPPH assay (%) (at 3–5 mg/mL)	60–65	*E. coli* *K. pneumoniae*	3.12–1.56 12.5–3.12	24.5–24.2 24.5–22.0	[53]
		β-carotene bleaching method (%) (at 1 mg/mL)	93–64	*S. enterica* *Enterobacter* sp.	6.25–0.78 12.5–3.125	18.5–19.02 2.7–17.5	
		Metal chelating activity IC_50_ (µg/mL)	250–367	*M. luteus*	12.5–3.12	44.5–43	
				*S. aureus* *B. cereus*	6.25 6.25	17.7–18 11.5–19.0	
Sulfated polysaccharides	Common smooth hound (*Mustelusmustelus*) 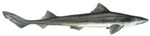	Ferrous chelating effect IC_50_ (µg/mL)	25.04–34.07	*S. aureus* *M. luteus*	- -	7.0–11.5 27.0–31.0	[54]
				*B. cereus* *E. coli*	- -	8.2–14.5 10.2–16.5	
		β-carotene bleaching inhibition (%) (at 0.25 mg/mL)	52–83	*K. pneumonia* *S. enterica*	- -	30.5–31.0 9.5–12.5	
		DNA nicking assay (at 50 and 100 µg/mL)	-	*S. typhi* *Enterobacter* sp.	- -	20.5–26.5 7.5–14.5	
Sulfated polysaccharides from *Pleurotu seryngii* (PEPS) and *Streptococcus thermophilus ASCC 1275* exopolysaccharides (ST1275 EPS)	*Streptococcus thermophilus ASCC 1275*	DPPH assay (%) at 1000 µg/mL	14.55–7.71	*S. aureus*	<0.625–2.5	14.5–31.8	[28]
	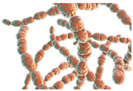	Superoxide radical scavenging activity (%) at 1000 µg/mL	35.10–22.33	*E.coli*	2.5–5.0	9.8–11.7	
	Sulfated *Pleurotu seryngii*						
	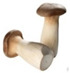	Hydroxyl radical scavenging activity (%) at 1000 µg/mL	23.44–21.81	*L. monocytogenes*	1.25–10.0	9.8–17.3	
Exopoly- saccharide (EPS) isolated from *Lactobacillus plantarum* (EPLB)	*Lactobacillus plantarum* 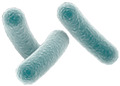	DPPH assay IC_50_ (mg/mL)	0.59–0.17	*S. aureus*	2	-	[55]
		Linoleic acid peroxidation with TBARS assay IC_50_ (mg/mL)	0.57	*L. monocytogenes*	10	-	
				*P. aeruginosa*	1	-	
				*S. typhymurium*	2	-	

DPPH—2,2-diphenyl-1-picrylhydrazyl; FRAP—ferric reducing antioxidant power; TBARS—thiobarbituric acid reactive substances; *Escherichia coli (E. coli); Pseudomonas aeruginosa (P. aeruginosa); Bacillus subtilis (B. subtilis); Staphylococcus aureus (S. aureus); Micrococcus luteus (M. luteus); Salmonella enterica (S. enterica); Klebsiella pneumonia (K. pneumonia)*; *Salmonella typhi* (*S. typhi*); *Salmonella typhimurium (S. typhimurium); Bacillus megaterium (B. megaterium); Listeria monocytogenes (L. monocytogenes); Aeromonas hydrophila (A. hydrophila); Enterococcus faecalis* (*E. faecalis)*.

**Table 2:** Application of polysaccharides as a natural preservative in meat and meat products.

Active Compound	Sample	Added Levels	Storage Conditions	Effect	References
Polysaccharides from pistachio external hull (PHCP)	Minced beef meat	0.5%, 1%, and 2% to 20 g of ground meat	9 days at 4 °C	Inhibited lipid oxidation (TBARS production). Improved the stability of meat color.	[95]
Polysaccharides from garlic straw (GSP)	Minced beef meat	2%, and 4% to 25 g of minced beef meat	9 days at 4 °C	Protected ground beef against lipid peroxidation. Increased shelf life. Improved sensory attribute (color).	[96]
Sulfated exopolysaccharides from *Porphyridium cruentum* (EPS)	Minced beef meat	0.5%, 1%, and 2% (equivalent to MIC, 2 × MIC and 4 × MIC against *L. monocytogenes* ATCC19117, respectively.)	plastic vacuum bags, 14 days at 4 °C	Extended the shelf life of ground beef by inhibiting the spoilage microorganisms. Prevented lipid and protein oxidation of minced meat.	[97]
Exopolysaccharide produced by *Lactobacillus* sp. Ca6 (EPS-Ca6)	Beef sausage	Vit C at 0.0625% + EPS-Ca6 at 0.0625%, and EPS-Ca6 at 0.125%	12 days at 4 °C	Retarded lipid peroxidation during refrigerated storage. Reduced the oxymyoglobin oxidation.	[96,97,98,99]
Polysaccharides derived from green seaweed “*Chaetomorpha linum*” (PS)	Beef sausage	0.05%, 0.125%, 0.25%	12 days at 4 °C	Increased pH and moisture values. Improved color stability. Stabilized MetMb and heme iron values. Decreased lipid oxidation. Reduced microbial counts.	[100]
Polysaccharides from *Trigonella foenum-graecum* (FWSP)	Beef sausage	0.05%, 0.125%, 0.25%	10 days at 4 °C	Reduced meat lipid oxidation. Significantly inhibited myoglobin oxidation.	[101]
A water-soluble polysaccharide from *Anethum graveolens*	Turkey meat sausages	0.05%, 0.15%, 0.3%.	polyethylene bag for 12 days at 4 °C	Increase the humidity level. Reduced lipid peroxidation. Preserved pH and color. Extended the shelf life by minimizing the growth rate of several bacteria.	[102]
*Spirulina platensis* polysaccharides (SPP)	Chinese-style (pork) sausages	0.1%, 0.25%, 0.5%	24 days at 4 °C	Maintained stable redness values. Preserved pH. Prevented the decrease in aroma, flavor, and sensory acceptance. Decreased lipid peroxidation.	[103]

Main effects of the addition of polysaccharides as a natural preservative in meat and meat products, according to the sources of polysaccharides, the percentage added and the storage conditions.

## Data Availability

Not applicable.
